# Improving the Gene Ontology Resource to Facilitate More Informative Analysis and Interpretation of Alzheimer’s Disease Data

**DOI:** 10.3390/genes9120593

**Published:** 2018-11-29

**Authors:** Barbara Kramarz, Paola Roncaglia, Birgit H. M. Meldal, Rachael P. Huntley, Maria J. Martin, Sandra Orchard, Helen Parkinson, David Brough, Rina Bandopadhyay, Nigel M. Hooper, Ruth C. Lovering

**Affiliations:** 1 UCL Institute of Cardiovascular Science, University College London, Rayne Building, 5 University Street, London WC1E 6JF, UK; barbara.kramarz@ucl.ac.uk (B.K.); r.huntley@ucl.ac.uk (R.P.H.); 2 European Bioinformatics Institute (EMBL-EBI), European Molecular Biology Laboratory, Wellcome Genome Campus, Hinxton, Cambridge CB10 1SD, UK; paola@ebi.ac.uk (P.R.); bmeldal@ebi.ac.uk (B.H.M.M.); martin@ebi.ac.uk (M.J.M.); orchard@ebi.ac.uk (S.O.); parkinson@ebi.ac.uk (H.P.); 3 Division of Neuroscience and Experimental Psychology, School of Biological Sciences, Faculty of Biology, Medicine and Health, Manchester Academic Health Science Centre, University of Manchester, AV Hill Building, Oxford Road, Manchester M13 9PT, UK; david.brough@manchester.ac.uk (D.B.); nigel.hooper@manchester.ac.uk (N.M.H.); 4 UCL Queen Square Institute of Neurology and Reta Lila Weston Institute of Neurological Studies, 1 Wakefield Street, London WC1N 1PJ, UK; rina.bandopadhyay@ucl.ac.uk

**Keywords:** Alzheimer’s disease, dementia, cognitive impairment, neurodegeneration, Gene Ontology, annotation, biocuration, amyloid-beta, microtubule-associated protein tau

## Abstract

The analysis and interpretation of high-throughput datasets relies on access to high-quality bioinformatics resources, as well as processing pipelines and analysis tools. Gene Ontology (GO, geneontology.org) is a major resource for gene enrichment analysis. The aim of this project, funded by the Alzheimer’s Research United Kingdom (ARUK) foundation and led by the University College London (UCL) biocuration team, was to enhance the GO resource by developing new neurological GO terms, and use GO terms to annotate gene products associated with dementia. Specifically, proteins and protein complexes relevant to processes involving amyloid-beta and tau have been annotated and the resulting annotations are denoted in GO databases as ‘ARUK-UCL’. Biological knowledge presented in the scientific literature was captured through the association of GO terms with dementia-relevant protein records; GO itself was revised, and new GO terms were added. This literature biocuration increased the number of Alzheimer’s-relevant gene products that were being associated with neurological GO terms, such as ‘amyloid-beta clearance’ or ‘learning or memory’, as well as neuronal structures and their compartments. Of the total 2055 annotations that we contributed for the prioritised gene products, 526 have associated proteins and complexes with neurological GO terms. To ensure that these descriptive annotations could be provided for Alzheimer’s-relevant gene products, over 70 new GO terms were created. Here, we describe how the improvements in ontology development and biocuration resulting from this initiative can benefit the scientific community and enhance the interpretation of dementia data.

## 1. Introduction

Understanding the cellular bases of Alzheimer’s disease (AD) and other dementias is an essential step in helping develop improved therapies for their treatment and prevention, as well as supporting early diagnosis. Although several genes associated with monogenic AD have been identified [1], the majority of cases are likely to be due to multiple genetic, as well as environmental, risk factors [2,3]. In order to understand the cellular processes and risk factors associated with AD and other dementias, numerous transcriptomic, proteomic, and genome-wide association (GWA) studies have been conducted [2,4,5,6]. Analyses and the interpretation of such high-throughput datasets rely on high-quality annotations describing the biological roles of the implicated gene products, as well as appropriate methodologies being used to generate the data, the application of appropriate statistical methods, and implementation in analysis tools [4,6,7,8]. Gene annotation provides functional knowledge of gene products (proteins and RNAs) in a format that can be fully exploited by systems biology and genomic investigators. Thus, annotation resources bridge the gap between data collection and data analysis [4,9]. The main resources used to identify significantly enriched pathways in GWA, proteomic, and transcriptomic studies are those provided by the Gene Ontology (GO) [10], Reactome [11], KEGG [12], and molecular interaction databases [13,14]. This data is imported into independent enrichment analysis tools, such as g:Profiler [15], VLAD [16], or DAVID [17], which can provide different enrichment options and/or settings, and are updated with different frequencies, all of which will affect analyses’ outcomes.

**Figure 1:**
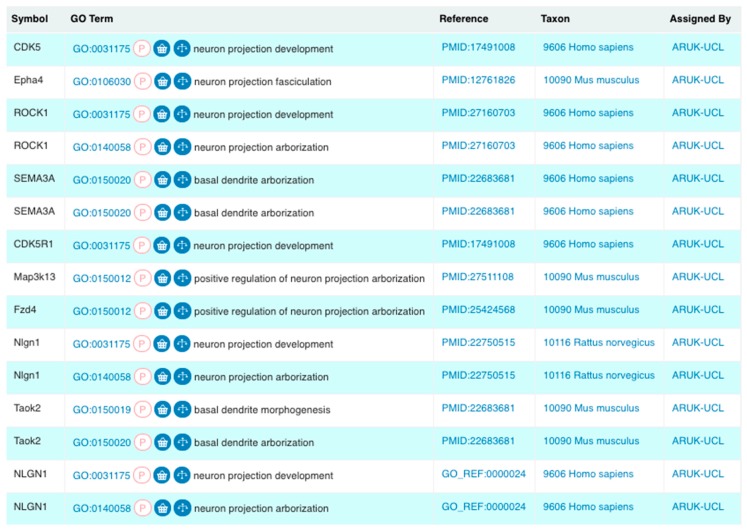

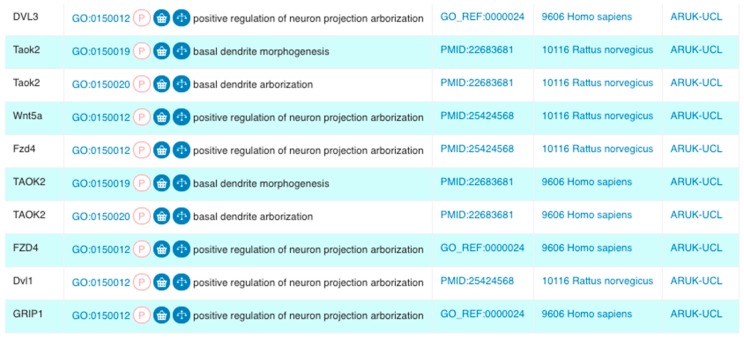
A selection of Gene Ontology (GO) annotations created by Alzheimer’s Research United Kingdom (ARUK)—University College London (UCL), as a part of our project that focused on capturing knowledge on amyloid-beta and tau by the expert biocuration of experimental data available through the biomedical literature. These annotations describe related biological processes using GO terms, which are descendants of the ‘neuron projection development’ GO term. We created 13 and revised two of the 24 GO terms in this branch. We created a total of 50 new annotations using these new neurological GO terms (Results sections: Revisions and development of neuron projection GO branches; Figure 4). ‘Symbol’ corresponds to the HUGO Gene Nomenclature Committee (HGNC)-approved gene symbol [20] encoding the gene product being annotated; the ‘GO Term’ section provides the GO identifier and term name; the ‘Reference’ column lists the source of the data supporting the annotation; this may be the curated article or information about the electronic annotation pipeline. The ‘Taxon’ column describes the species of origin of the protein being annotated; and the ‘Assigned By’ column shows that these annotations resulted from this project (Screenshot from QuickGO—accessed: 31 October 2018).

The GO resource is a biomedical ontology that describes the physiological functional attributes of gene products, across all species, in a consistent and computer-accessible manner using a controlled vocabulary that facilitates the integration of public biological data [10,18,19]. Fully defined GO terms are associated with gene products across many species, providing a computable summary of individual experiments that demonstrate functional information (Figure 1). These links between GO terms and gene products are known as ‘annotations’, and enable the description of gene products according to their molecular functions (e.g., ‘scavenger receptor activity’), the biological processes they contribute towards (e.g., ‘microtubule cytoskeleton organisation’), and their subcellular locations (or cellular components, e.g., ‘extracellular region’) [10].

One of the key advantages of the GO resource is that it can describe a protein’s likely role in a process or its probable location in a cell, even when its actual functional roles are still under investigation. For instance, GO will capture the role of a protein kinase in a signalling cascade even when the step in this cascade at which the enzyme is acting has not yet been fully elucidated. In contrast, Reactome and KEGG provide more ‘specific’ information about the role of a protein within a pathway by creating networks where the details of each ‘reaction’ being catalysed or facilitated are provided [11,12]. Consequently, proteins whose roles have not been fully elucidated cannot be included in these databases. However, occasionally Reactome will capture ‘BlackBox’ events when not all of the steps in a pathway are known [11]. In summary, Reactome provides annotations for 10,800 human proteins, whereas GO provides manual annotations for 20,000 human proteins (data from QuickGO and Reactome, accessed: 20 August 2018). A common strength of all of these resources is that for any curated model organism, data can be conservatively mapped across to their human (and other species) orthologs; however, because many gene products are involved in multiple cellular processes, the comprehensive annotation of all gene products has not been achieved. Furthermore, although human and mammalian phenotype ontologies (HPO, MP) are being used to interpret next generation sequencing (NGS) data [21], understanding how multiple genes contribute to a single disease or phenotype will require resources, such as GO, that describe the cellular functions of these gene products.

Amyloid-beta fibril deposits commonly known as plaques, and neurofibrillary tangles of phosphorylated tau, have for decades been the pathological hallmarks of Alzheimer’s disease [22]. For many years, researchers have been studying the formation and the effects of these protein aggregates on brain function [23,24,25,26]. However, much less attention and fewer resources have been committed towards elucidating the underlying cellular mechanisms that drive the formation of these protein aggregates, or towards understanding the roles of the various cell types involved in the maintenance of normal brain functions, including not only neurons, but also glia and the cells of the brain microvasculature [27,28,29]. It is currently commonly accepted within the dementia research communities that impairments in cellular processes that lead to disease onset can occur decades before the first manifestations of clinical symptoms [27]. Accordingly, the research focus has been steadily shifting from protein aggregates towards protein homeostasis and the implicated cellular processes [30]. This has coincided with the ‘explosion’ of ‘big data’ in biomedicine. GWA and biomarker expression studies have resulted in the generation of extensive datasets of gene loci linked to dementia and AD [31,32,33,34,35]. In order to reveal the biological processes that are impaired in dementia due to changes in expression levels and/or the regulation of these genes, high-throughput datasets can be interpreted using GO term enrichment analysis or pathway analysis. By using the contextual information available in GO, it would also be possible to identify the cell type(s) in which an enriched process most likely occurred, if gene expression had been measured across the whole brain rather than in specific cell types or individual cells [35]. Identification of the key cells that are involved is also likely to contribute to our understanding of the cellular mechanisms underlying AD. For example, Espuny-Camacho et al. performed GO gene set enrichment analysis on a novel chimeric model of AD, and observed an increased expression of genes involved in myelination, and a decreased expression of genes related to memory and cognition, synaptic transmission, and neuron projection [36]. Furthermore, two recent studies have successfully used GO to interpret AD patient data. Xu et al. [37] investigated regional protein expression in the brain of human AD patients, and showed the involvement of some of the differentially expressed genes in key neurological processes, such as ‘nervous system development’ (GO:0007399) and ‘neuron projection development’ (GO:0031175). Kunkle et al. [38] described GWA data from the largest cohort to date of late-onset AD cases, and demonstrated the involvement of the differentially expressed genes in dementia-relevant processes such as ‘regulation of amyloid-beta formation’ (GO:1902003), ‘tau protein binding’ (GO:0048156) and ‘activation of immune response’ (GO:0002253). Another study applied a new statistical approach that used GO with neuroimaging phenotypes and GWA data to identify gene products that are involved in processes impaired in disease [39]. A peculiarity of that work was the inclusion of a focussed GO annotation of 21 gene products, the majority of which were encoded by AD-associated Mendelian genes. The 400 manual annotations that the authors associated with these and eight other genes were included in GO annotation files prior to carrying out the analysis. Last but not least, GO can also be used in the reanalysis of previously published datasets, which is a potentially productive exercise, because the availability of new GO data impacts on the information content [40].

In order to meet the urgent requirement to improve the interpretation of and draw informative conclusions from high-throughput dementia studies, the focus of the project described here was to provide GO annotations that capture knowledge on amyloid-beta and tau by the expert biocuration of experimental data available through the biomedical literature (annotations resulting from this project are assigned by ‘ARUK-UCL’). We have now completed the first phase of enhancing the GO resource by developing new neurological GO terms and capturing the roles of proteins interacting with amyloid-beta and tau using functional GO annotations. The curation of 40 amyloid-beta binding proteins (i.e., proteins and protein complexes acting as amyloid-beta receptors [41]) has provided over 2000 annotations describing the normal role of these proteins in signalling, receptor-mediated endocytosis, and phagocytosis and/or clearance of amyloid-beta. In addition, the normal roles of microtubule-associated protein tau and its interacting partners [42] are now also captured in GO annotation files, following the annotation of 33 proteins and the creation of over 1700 annotations. Here, we provide summaries of key areas of improvement, as well as an in-depth discussion of the specific challenges that arose during this work.

## 2. Materials and Methods

### 2.1. Community Involvement

Collaborations have been established between members of the Gene Ontology Consortium [43] and community experts to ensure that our biocuration efforts aligned with the needs of the neuroscience research community. Project progress and direction were discussed and, if required, revised and updated during project meetings and through regular correspondence.

### 2.2. Selection of Experimental Data to Annotate

Two sets of AD-relevant high-priority human proteins and protein complexes were compiled based on recent review articles. The first set included amyloid-beta species and their binding partners, which have been shown to act as receptors for the amyloid-beta monomers and/or their oligomers, and were fully based on the amyloid-beta receptors listed in the Jarosz-Griffiths el al. (2016) review [41]. The second set was a collection of proteins interacting with the microtubule-associated protein tau, based on the review article by Guo, Noble, and Hanger (2017) [42]. This review provided a vast list of tau-binding partners, of which 33 were selected for annotation after consultations with the neuroscience community. Primary research articles cited in both reviews were prioritised for annotation. The PubMed database [44] was also used to identify additional research articles that contained experimental data describing each of the human gene products prioritised for annotation. For each high priority protein/protein complex, PubMed searches were performed using the gene symbols and names approved by the HUGO Gene Nomenclature Committee (HGNC) [20], as well as their synonyms. For well-researched gene products, relevant papers were identified with secondary searches for gene symbols and names combined with the following keywords (one at a time): ‘dementia’, ‘Alzheimer’s’, ‘Alzheimer’, ‘AD’, ‘amyloid’, ‘APP’ (amyloid precursor protein), ‘tau’, ‘*MAPT*’ (microtubule-associated protein tau), ‘microtubule’, ‘neurology’, ‘neurological’, ‘neurobiology’, or ‘neurodegeneration’. If no, or insufficient, information on the human gene product was found, orthologs, identified using the HGNC ortholog prediction tool ‘HCOP’, were curated [20]. Peer-reviewed articles were selected for curation based on criteria described by Denny et al. [40], i.e., (1) they contained experimental research data; (2) the curation of any given article would result in new information being added to the current GO annotation data associated with the prioritised gene products; (3) it was possible to identify the species from which the gene products and/or expression constructs were derived, crucial information enabling biocurators to assign the appropriate database identifiers to gene products. Selected research articles were annotated fully, resulting in GO annotations of not only the dementia-relevant high-priority gene products, but also any other proteins or protein complexes described in those articles.

### 2.3. Gene Ontology Annotation of Proteins and Protein Complexes—Manual Curation Process

Primary research articles were read by skilled GO biocurators to describe the molecular functions, biological processes, and cellular locations of the gene products and capture them using GO terms, following established GO Consortium guidelines [40,45]. In addition, often terms from other ontologies were included in the GO annotation extension field to provide additional contextual information [46]. The gene product identifiers that were used in this project included UniProt accessions [47], Complex Portal accessions [14], as well as RNAcentral accessions [48]. Specific evidence codes were included in each biocurator-generated annotation, based on the type of experimental data reported in the research articles (e.g., IPI: inferred from physical interaction, or IMP: inferred from mutant phenotype), or to infer evidence from statements made in reviews (e.g., TAS: traceable author statement [49]). GO annotations created for rodent or other mammalian gene products, based on experimental evidence, were transferred by biocurators to human orthologs using the evidence code ISS (inferred from sequence similarity), if 1-to-1 orthology could be confirmed. This biocuration process was consistently applied to describe the published experimental data for each of the human high-priority genes and/or their orthologs. The annotations contributed by this project to the GO resource are attributed to ARUK-UCL, and were captured using the European Bioinformatics Institute (EBI) GO annotation tool Protein2GO [50]. All of the ARUK-UCL annotations are included in the GO Consortium annotation files, and thus made available through various ftp sites and the GO browsers QuickGO [51,52,53] and AmiGO [54,55], which are updated on a weekly and monthly basis, respectively. Our GO annotations are also propagated to other major biological databases, including NCBI Gene [56], Ensembl [57], UniProt [47], RNAcentral [48], and miRBase [58]. Additionally, the GO annotation work that was completed as a part of this project contributed to the biocuration of new Complex Portal [14] entries.

### 2.4. Ontology Development and Integration of Resources

The ontology editor Protégé [59] was used to generate new GO terms and, if required, modify and/or update the existing terms. All of the ontology changes were integrated into the public ontology version using GitHub [60]. In the QuickGO browser [51,52,53], GO term entries that were created and/or updated as a result of this project include an acknowledgment for ARUK. In the AmiGO browser [54,55], the same GO terms are identified by the source ‘GOC:aruk’ [61].

### 2.5. Functional Analysis of Hippocampal Proteomic Data

To identify the over-representation of GO terms in a hippocampal protein dataset (Table S8), which was identified as differentially expressed in Alzheimer’s disease patients compared to age-matched controls [37], the functional analysis tool g:GOSt available from the g:Profiler server [15] was used. The analysis was undertaken on 19 November 2018, using the annotated human proteome as the background ‘population’ set. The g:SCS method was used for computing multiple testing correction for the GO enrichment analysis *p*-values, with an experiment-wide threshold of a = 0.05. This algorithm considers the ontology structure underlying the gene sets annotated to each GO term. The analysis used two g:GOSt preloaded GO datasets: December 2016 includes the annotation file available in Ensembl 87 (build date December 2016) and the ontology file released 13 December 2016; November 2018 includes the annotation file available in Ensembl 93 (build date July 2018) and ontology file released 3 August 2018. All of the protein identifiers (Table S8) were provided by Xu et al. [37]. In addition, this dataset was also ‘seeded’ with the full list of priority proteins, in order to further examine the impact of this annotation project; this list is subsequently referred to as the ‘priority protein-seeded hippocampal’ list.

## 3. Results

### 3.1. Assignment of Database Identifiers

The gene products that were prioritised for Gene Ontology (GO) annotation were identified from two key reviews [41,42] and finalised following consultations with dementia experts. Forty-nine gene products implicated in amyloid-beta biology were prioritised for annotation; this list included 40 cellular receptors reviewed by Jarosz-Griffiths et al. [41], to which nine monomeric and oligomeric amyloid-beta species have been shown to bind. Among the tau-interacting partners, 33 were prioritised for annotation based on the review by Guo, Noble, and Hanger [42]; the priority list therefore includes 34 proteins (also counting tau itself). The focus of this project was the annotation of human gene products; therefore, all of the 84 prioritised entities (76 proteins and eight complexes) are referred to using database identifiers corresponding to human gene products (Table S1).

In order to capture amyloid-beta biology accurately, it was necessary to be able to distinguish between amyloid-beta monomers, dimers, and oligomers, because of the different cellular effects of monomeric amyloid-beta and its dimeric or oligomeric forms [62]. Consequently, a collaboration was established with Complex Portal (CP) [14,63] biocurators, which resulted in the generation of 18 new CP entries for the different amyloid-beta monomers, dimers, and oligomers in three species (human, rat, and mouse) (Table S2).

### 3.2. Gene Ontology Annotation

Research articles were selected as described in the methods section and annotated following the established GO guidelines for manual biocuration [40]. Of the 226 PubMed-indexed articles that we curated to capture knowledge about our 84 prioritised gene products, 191 described roles of human gene products (Table 1, Table S3C). Twenty-five articles that had appeared in the PubMed searches and had been read were deemed not suitable for annotation; 12 of these provided no information about the species of the gene product(s) being annotated; six provided information about either disease, patients, or pharmacological agents; three provided expression data without functional information; and three were either opinion or review articles; whereas one article contained data images of insufficient quality for a biocurator to be able to review and capture the experimental information in a GO annotation(s) (Table S3D). Gene products were associated with relevant GO terms according to evidence provided in the published research articles. Rather than capturing the experimental data describing only the gene products on the priority list, all of the experimental data in each curated paper was associated with GO terms, thus increasing the number of gene products curated, which is an efficient approach that is often referred to as ‘full paper curation’.

Through our whole-paper curation approach, a total of 3886 GO annotations have been contributed for a total of 561 gene products, including proteins (UniProt [47,64] identifiers), microRNAs (RNAcentral [48] identifiers), and protein complexes (Complex Portal [14,63] identifiers) as a part of this ARUK-UCL annotation initiative. Among these, 2770 GO annotations were associated with 308 human gene products, and 2055 were associated specifically with the proteins and complexes prioritised for annotation (Table 1, Tables S1–S4); based on data from QuickGO, accessed 17 August 2018).

**Figure 2:**
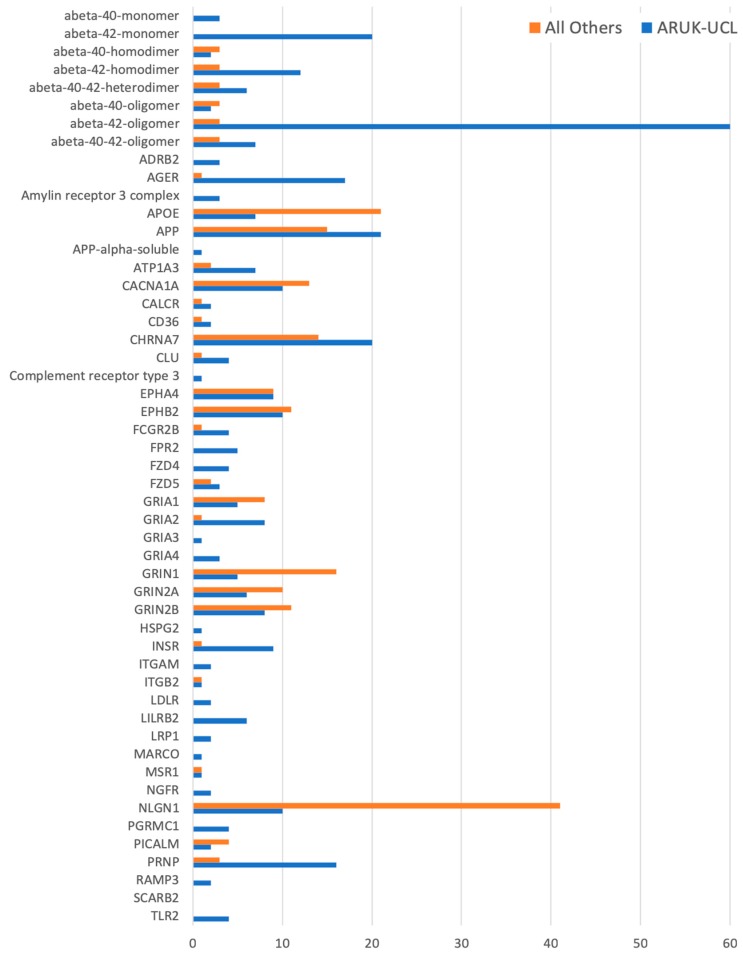
A histogram of all of the manually curated neurological GO annotations for the prioritised amyloid-beta-relevant human gene products, including annotations contributed by ARUK-UCL as well as by other groups. Data for this figure was derived from Table S5.

**Figure 3:**
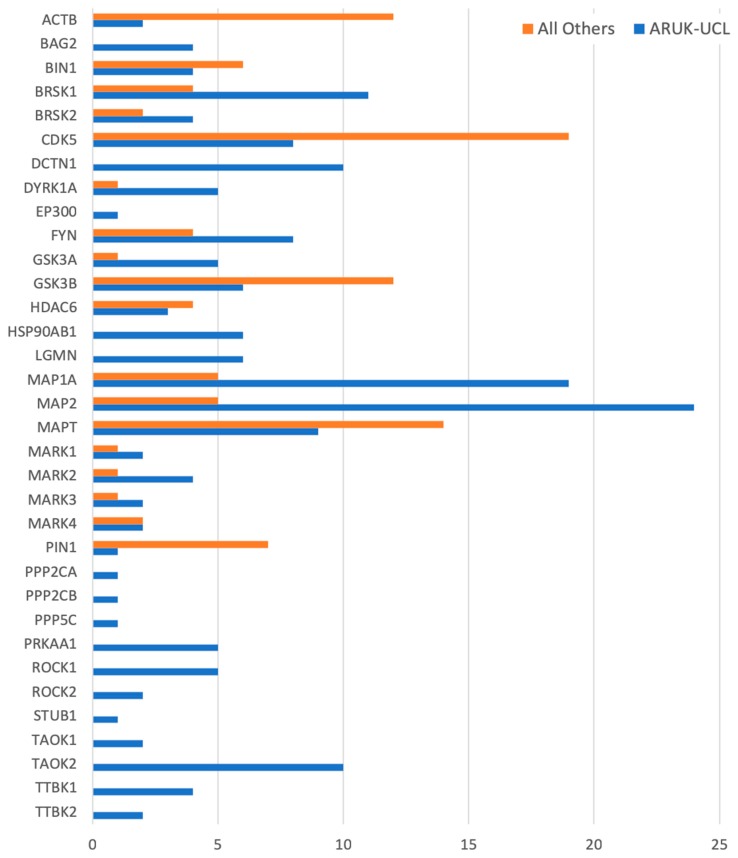
A histogram of all the manually curated neurological annotations for the prioritised tau-relevant human gene products, including annotations contributed by ARUK-UCL as well as by other groups. Data for this figure was derived from Table S5.

The annotations that we have provided ensure that the role of these gene products in dementia-relevant processes is captured; overall, this project created just over a quarter of the annotations now associated with these 84 protein and complex records. For example, several of the priority genes (such as *APP*, *APOE*, *FYN*, and *HSP90AB1*) had over 200 associated GO annotations prior to the start of this project, but few of these described neurological processes. Our work has resulted in 526 new annotations to neurological GO terms for all but one of the prioritised gene products (Figure 2 and Figure 3, Table S5), capturing their dementia-relevant roles in neurological processes. For one, *SCARB2*, no evidence to support the association of a neurological or amyloid-related GO term was identified in the published literature.

Overall, approximately two-thirds (526 of the 834) of the neurological, amyloid-beta, or tau-relevant GO terms, which are now associated with 84 of our prioritised gene products, were created by the ARUK-UCL initiative. Previously, only 49 of these gene products were associated with neurological process, a neuronal cellular location, or a molecular function relevant to amyloid-beta or tau biology (Figure 1 and Figure 3, Table S5). In summary, the ARUK-UCL contribution greatly increased the number of our prioritised gene products being associated with neurological process GO terms, e.g., ‘amyloid-beta clearance’ and ‘learning or memory’, as well as localising to neuronal structures, including axons, dendrites, and their compartments.

### 3.3. Gene Ontology Development and Revisions

#### 3.3.1. New and Revised Gene Ontology Terms

As the literature curation progressed, new GO terms were needed to represent normal cellular functions or compartments altered in dementia. In some cases existing GO terms were also revised and updated with recent knowledge, or with synonyms that were useful for biocuration and literature mining (Table S6). For instance, 10 new GO terms have been added to the ‘neuron projection morphogenesis’ biological process branch, and numerous corresponding ‘neuron projection’ cellular component terms have been revised. In addition, new molecular function and biological process terms have been added to lipoprotein particle-related terms. Moreover, revisions have been made to ‘synaptic vesicle endocytosis’, and new child terms, for this GO term, have been added. Overall, 84 GO term entries were curated as part of this project; of these, 71 new GO terms were created, and 13 existing GO terms were modified (Table S6). Sixty-eight of these new, or modified, GO terms represent biological processes; 15 are cellular component terms, and one is a molecular function term. These terms were used to curate proteins and protein complexes prioritised for annotation (Table S1) due to their association with dementia. Details of these new additions and revisions can be tracked on the GitHub Gene Ontology pages [60] by filtering issues marked with ‘ARUK-UCL’ labels.

#### 3.3.2. Revisions and Development of Neuron Projection Gene Ontology Branches

The revision and modification, as well as creation, of GO terms describing neuron projection-relevant processes are key contributions of this project, and were undertaken to improve the representation of dementia-relevant brain biology. Axons and dendrites, i.e., pre-synaptic and post-synaptic neuron projections, enable neuron-to-neuron signalling through synapses, whereas synaptic plasticity, which is dependent on the structural and functional integrity of synapses between neuron projections, is key for memory formation and learning [65]. Early in this project, the need to revise the ‘neuron projection’ GO domain was identified in order to capture full functional information that describes how the binding of amyloid-beta to receptors on pre-synapses and post-synapses or the presence of phospho-tau neurofibrillary tangles in synaptic compartments may lead to the modulation of synaptic plasticity [66,67].

**Figure 4:**
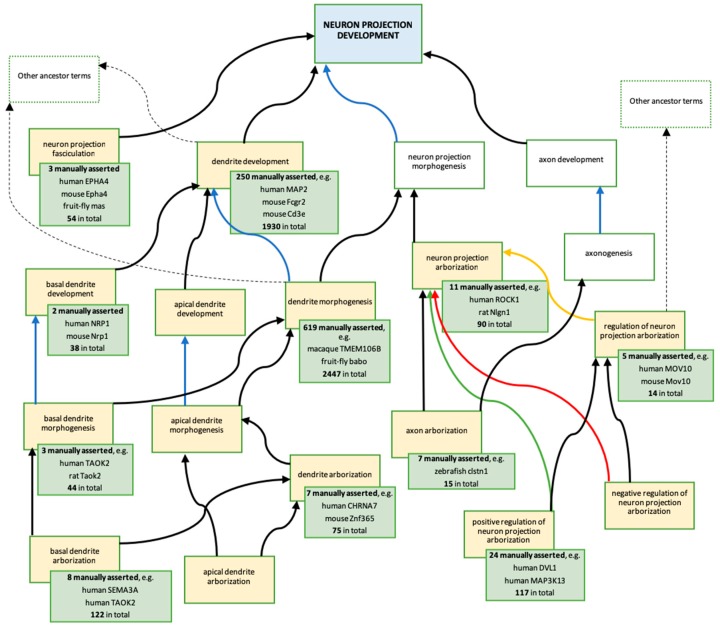
Descendants of biological process ‘neuron projection development’ GO term created or modified, as a result of this ARUK-UCL project. Ontology: The blue box represents the ancestor term ‘neuron projection development’ (GO:0031175). The yellow boxes represent the terms created, or modified, as a result of the ARUK-UCL project (Table S6); the majority of these are new terms; two terms were modified: ‘dendrite development’ (GO:0016358) and ‘dendrite morphogenesis’ (GO:0048813). The white boxes represent related terms in the ontology; no changes have been made to these terms. Arrows indicate the following ontology relationships: black—is_a, blue—part_of, yellow—regulates, green—positively_regulates, red—negatively_regulates. Annotations: Each green box provides information about gene products associated with the GO term in the yellow box, which it overlaps (data from QuickGO [51,52,53], accessed: 20 November 2018). For total numbers of annotations, provided in the green boxes, the following QuickGO filter was used: ‘GO terms → Options → Use these terms as an exact match’; an additional filter ‘Evidence → ECO:0000352: evidence used in manual assertion’ was then applied to retrieve the numbers and examples of manually asserted annotations; these include annotations contributed by ARUK-UCL and by other groups. Examples of annotations including GO terms shown above are presented in Figure 1.

Literature curation did confirm the involvement of many of the prioritised gene products (Table S1) in ‘neuron projection development’ and ‘neuron projection organisation’. This resulted in 33 experimental, or orthology-based, GO annotations applying terms descended from ‘neuron projection development’ (Figure 1 and Figure 4); all of these annotations were made to the GO terms created, or revised, by ARUK-UCL. Biocurators from the Rat Genome Database (RGD) [68] also contributed to the development of this GO branch or terms descended from ‘neuron projection development’ by requesting regulation terms of ‘neuron projection arborisation’.

**Figure 5:**
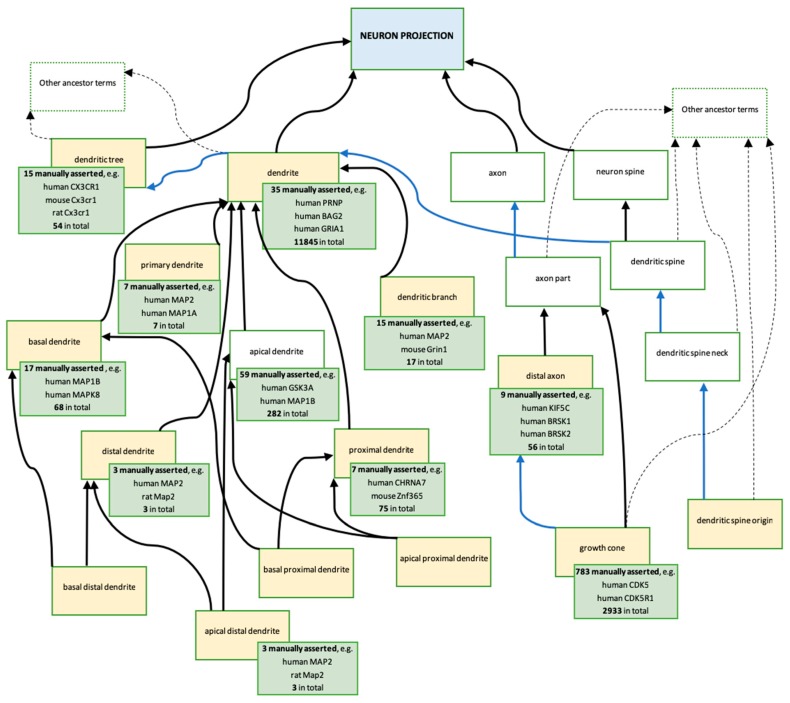
Descendants of cellular component ‘neuron projection’ GO terms created or modified, as a result of this ARUK-UCL project. Ontology: The blue box represents the ancestor term ‘neuron projection’ (GO:0043005). The yellow boxes represent the terms created, or modified, as a result of the ARUK-UCL project (Table S6). The majority of these are new terms; five terms were modified: ‘dendritic tree’ (GO:0097447), ‘dendrite’ (GO:0030425), ‘basal dendrite’ (GO:0097441), ‘dendritic branch’ (GO:0044307), and ‘growth cone’ (GO:0030426). The white boxes represent related terms in the ontology; no changes have been made to these terms. Arrows indicate the following ontology relationships: black—is_a, blue—part_of. Annotations: Each green box provides information about the gene products associated with the GO term in the yellow box, which it overlaps (data from QuickGO [51,52,53]—accessed: 20 November 2018). For the total numbers of annotations, which is provided in the green boxes, the following QuickGO filter was used: ‘GO terms → Options → Use these terms as an exact match’; an additional filter ‘Evidence → ECO:0000352: evidence used in manual assertion’ was then applied to retrieve the numbers and examples of manually asserted annotations. The green boxes include examples of gene products annotated by ARUK-UCL and other groups.

New additions and revisions of the biological process terms involving ‘neuron projection’ naturally led to revisions of the related cellular component terms (Figure 5). In parallel, revisions of the dendrite-related component terms were additionally prompted by RGD requests for new GO terms to describe ‘primary dendrite’, ‘distal dendrite’, and ‘dendritic spine origin’. This also led to a revision of the ‘dendritic tree’ (GO:0097447), ‘dendritic branch’ (GO:0044307), ‘dendrite’ (GO:0030425), and ‘basal dendrite’ (GO:0097441) terms; specifically, of their definitions and relations to other GO terms in the ontology. Among others, several of the prioritised gene products (Table S1) have been associated with descendants of the ‘neuron projection’ GO term (Figure 5, green boxes).

A major change to the cellular component ‘neuron projection’ branch, which resulted from the ARUK-UCL literature curation and RGD new term requests, concerned the conflicting definitions of ‘dendrite’ and ‘dendritic tree’, which previously stated that both of these neuron projections were branched, suggesting that both terms referred to the same structure. These definitions have now been modified in order to distinguish the terms from each other. The first sentence in the ‘dendrite’ definition has been changed from ‘a neuron projection that has a short, tapering, often branched morphology…’ to ‘a neuron projection that has a short, tapering, morphology…’. Whereas, the ‘dendritic tree’ definition now includes the sentence, ‘the entire complement of dendrites for a neuron, consisting of the primary dendrite and all its branches’. In addition, it was also necessary to revise the relationships between the different descendants of ‘dendritic tree’ as well as with its parent terms (Figure 5). The GO term ‘dendritic tree’ now has the is_a parent ‘neuron projection’, and the part_of parent ‘somatodendritic compartment’, and the direct is_a parent ‘cell part’ has been removed. In contrast, the term ‘somatodendritic compartment’ is no longer a direct part_of the parent of ‘dendrite’, which instead has the is_a parent ‘plasma membrane-bound cell projection part’ and the part_of parent ‘dendritic tree’.

**Figure 6:**
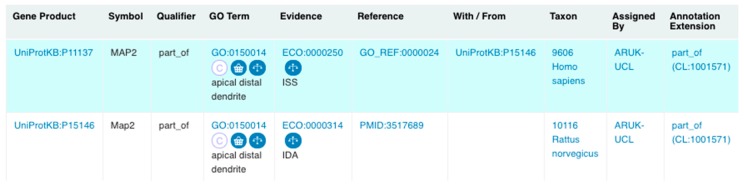
Rat Map2 and human MAP2 GO annotations to ‘apical distal dendrite’ (GO:0150014) assigned by ARUK-UCL. (Screenshot from QuickGO [51,52,53]—accessed: 31 October 2018).

Furthermore, as a result of the ARUK-UCL literature curation, even more specific ‘dendrite’ terms were added, including ‘apical distal dendrite’ (GO:0150014), ‘apical proximal dendrite’ (GO:0150015), ‘basal distal dendrite’ (GO:0150016), and ‘basal proximal dendrite’ (GO:0150017). The creation of these new terms was prompted by the necessity to capture the specific neuronal localisation of the MAP2 gene product, which was prioritised for annotation of tau-relevant biology (Table S1), to ‘apical distal dendrite’ (Figure 6). The RGD also requested the new term ‘distal axon’ (GO:0150034), which led to revisions of the axonal ‘neuron projection’ GO branch and resulted in the modification of definition and relations of ‘growth cone’ (GO:0030426) (Figure 5).

#### 3.3.3. Concerted Effort of GO Consortium Members: Revisions of ‘Synaptic Vesicle Endocytosis’

The ‘synaptic vesicle endocytosis’ GO term was originally defined as dependent on clathrin; however, not all synaptic vesicle endocytosis is clathrin-mediated [69,70]. Some of the GO annotations that had been made to ‘synaptic vesicle endocytosis’ may not have been based on clathrin-mediated mechanisms; the gene products may have been involved in an alternative mechanism of ‘bulk synaptic vesicle endocytosis’. Consequently, GO biocurators agreed to review 97 experimentally evidenced annotations to ‘synaptic vesicle endocytosis’ (GO:0048488) and its child terms, including ‘(positive/negative) regulation of synaptic vesicle endocytosis’ terms. At least a third of the ‘synaptic vesicle endocytosis’ annotations lacked experimental evidence that confirmed the involvement of clathrin in the curated data. There was unanimous agreement among the GO contributors that the definition should be made more general, in order to encompass the mechanisms of synaptic vesicle import independent of clathrin. Additionally, in order to allow for more specific annotations, we created two new child terms of ‘synaptic vesicle endocytosis’ (GO:0048488): ‘clathrin-dependent synaptic vesicle endocytosis’ (GO:0150007) and ‘bulk synaptic vesicle endocytosis’ (GO:0150008). These terms are distinguished by their definitions: GO:0150007: ‘Clathrin-dependent endocytosis of presynaptic membrane regions comprising synaptic vesicles’ membrane constituents. This is a relatively slow process occurring in the range of tens of seconds.’; and: GO:0150008: ‘Endocytosis of large regions of presynaptic membrane after intense stimulation-mediated fusion of multiple synaptic vesicles. Bulk endocytosis is triggered by high loads of membrane addition through exocytosis of synaptic vesicles and elevated concentration of calcium in the presynapse’.

#### 3.3.4. Challenges Related to Emphasis on ‘Normal’ Processes

The GO has been designed to capture only ‘normal’ processes, functions, and cellular locations. When annotating gene products relevant to a specific disease, this presents a major challenge, as it is necessary to determine what is ‘normal’. For the annotation of AD-relevant gene products, this was a significant hurdle, particularly when capturing the roles of amyloid-beta species, including monomers, dimers, and oligomers (Table S2). This annotation project recognised that ‘ageing’ (GO:0007568) is a normal physiological process and consequently teased out data that described the association of dementia with ageing and the involvement of amyloid-beta in AD pathology [71,72,73,74,75]. Furthermore, we decided that it was necessary to capture the evidence that amyloid-beta (as well as tau) has normal physiological roles at the synapse [66,67], and, consequently, the normal roles of amyloid-beta complexes have been annotated as part of this project [62,66,76]. GO annotations describing age-related effects associated with amyloid-beta dimers and/or oligomers were also captured [71,72,73,74,75].

On the other hand, the literature review also revealed that there are some types of amyloid-beta oligomers, specifically the so-called amyloid-beta globulomers [77,78] and amylospheroids [79,80], which have only ever been observed in pathological cases; consequently, their roles have not been captured in GO.

### 3.4. Impact of Improved GO Annotation on Data Analysis

In order to evaluate the impact of this annotation project on high-throughput data interpretation, we undertook a functional analysis (Tables S7–S10) of a hippocampal proteomic dataset [37]. This dataset identified the hippocampal proteins that were differentially expressed in AD versus age-matched controls (Table S8), and analysed using the current (November 2018) GO annotation and ontology files preloaded in g:GOSt [15] as well as archived files (December 2016).

The previous analysis by Xu et al. [37] had identified a variety of enriched pathways in this dataset, including innate immune response, carbohydrate metabolism, and a variety of specific signalling pathways, such as those involved in the regulation of apoptosis and cell cycle, and pathways leading to neurotransmitter synthesis. Both our 2016 and 2018 g:GOSt analyses also identified these processes, as well as other neurologically-relevant terms such as synaptic signalling and axonal transport (Table S7). By grouping the enriched GO terms into broad classes (Tables S9 and S10), it was apparent that the majority of the enriched terms were associated with transport (i.e., localisation, transport, vesicle and vacuole terms), as demonstrated, for instance, by annotations to microtubule-related terms (Table S7, filter on column A by ‘microtubule’), in addition to metabolism and the organisation and biogenesis of specific cellular components.

A comparison between the GO terms enriched using the 2016 versus the 2018 files (Table S11) demonstrates how the continued contribution of annotations to the GO Consortium resource and the revision of the ontology continues to impact the analysis of high-throughput datasets. The 2016 to 2018 comparison identified a difference between these analyses, with 84 terms identified only in the 2016 analysis, and 181 enriched GO terms only present in the 2018 analysis (Table S11A, column F, rows 5, 7, 14). Of particular note is the increase in the number of hippocampal proteins associated with immune system terms: using the 2016 GO files, only antigen presentation terms were identified as dysregulated in the hippocampus, while the 2018 analysis identified that 23% of dysregulated hippocampal proteins may be contributing to an immune response, with a suggestion that neutrophils are mediating this effect (Table S7, filter on column E by ‘immune system process’). Similarly, the GO term ‘neuron death’ is only enriched when using the 2018 GO files, with 4% of hippocampal proteins associated with this term (Table S7, filter on column E by ‘neuron death’).

To demonstrate the impact of the ARUK-UCL project on functional analysis (Tables S7–S10), we repeated the above analyses using the same hippocampal protein list, but with the addition of all the proteins that we had prioritised for annotation (the ‘priority protein-seeded hippocampal’ list). However, 10 of these proteins (CLU, DCTN1, FYN, GSK3A, GSK3B, HSP90AB1, HSPG2, PIN1, PPP2CA, and ROCK2) were present in both the hippocampal protein and priority protein lists (Table S8). While the same 633 GO terms can be seen as enriched in both the 2016 and 2018 analyses, for this ‘priority protein-seeded hippocampal’ list, there are 356 GO terms that are only enriched using the 2018 GO files (Tables S10 and S11). As the ARUK-UCL project has used many of the terms as only enriched in the 2018 analysis to capture the role of dementia-relevant gene products, it is likely that some of these differences can be attributed to this project. Notably, amyloid-beta clearance and neuroinflammation-relevant GO terms are enriched in the 2018 analysis, but not the 2016 analysis (Table S7, filter on column E by ‘amyloid-beta clearance’ and ‘neuroinflammation’). An investigation into the source of the ‘amyloid-beta clearance’ annotations associated with human proteins confirms that the ARUK-UCL project is responsible, either directly or indirectly (through the annotation of an ortholog), for all but one of these annotations (data from QuickGO—accessed: 20 November 2018). In addition, many of enriched GO terms that are identified only in the 2018 files with this ‘priority protein-seeded hippocampal’ list, are relevant to signalling and transport, reflecting the role of many of our priority proteins in these areas.

## 4. Discussion

Controlled biomedical vocabularies, or ontologies, have been used to semantically capture and describe AD-specific knowledge to enhance its sharing and exchange, as well as to aid collaborative funding and research efforts [81,82,83,84]. The use of ontologies has also been suggested to help with the prioritisation of genes for neuroimaging studies [39], as well as with the diagnosis of cognitive impairments [85]. With one exception [6], these vocabularies have been intended to capture and describe the domain of knowledge specific to disease, or phenotype, and not normal physiological processes.

Here, we used Gene Ontology (GO), a controlled biological vocabulary, to describe the normal roles of gene products implicated in AD and dementia. The advantage of GO is that it is a well-established and regularly maintained resource, which is commonly used for enrichment analyses across biomedical fields [40,86,87,88] and even in clinical practice [89]. In addition, during curation, we focus on a specific biological area, such as amyloid-beta receptors, and we annotate whole research articles using GO to capture the roles of all the gene products described. Thus, through our project, we enhanced GO more broadly, which not only benefits the Alzheimer’s disease researchers, but also the biomedical research communities overall.

### 4.1. Cellular Events Underlying Dementia

Amyloid-beta plaques and phospho-tau neurofibrillary tangles (NFTs) are the pathological hallmarks of dementias, and historically, they were believed to be the underlying causes of the dementia associated with AD [22,90,91,92]. It is now understood that in terms of amyloid-beta, AD pathology is exacerbated by its oligomers (amyloid-beta intermediates formed from the monomeric peptides prior to sedimentation into plaques) rather than the plaques, and the oligomers’ neurotoxicity depends on their concentrations and/or the presence of specific forms, such as globulomers or amylospheroids [71,72,73,74,75,77,78,79,80]. Whereas, phospho-tau has been demonstrated to contribute to dementia pathology due to its prion-like seeding activity [93,94,95,96], which allows the aberrantly phosphorylated tau aggregates to spread to as-yet unaffected brain regions, thus advancing the clinical symptoms. Yet, in recent years, scientific research has generated evidence, which demonstrates that the formation of these neurotoxic protein aggregates results from prior impairments in underlying cellular processes [27]. When these cellular brain processes are malfunctioning, this eventually leads to disruptions in protein homeostasis and the clinical manifestations of dementia [30]. Consequently, the plaques and/or NFTs that are characteristic of the brain pathology associated with advanced-stage dementia are currently believed to be the by-products of compensatory processes, which become activated in the brain at the cellular level to address the imbalances underlying the disease [30,97].

Therefore, until disease progression and its mechanisms are fully elucidated and understood, it should not be assumed that the fibrils of amyloid-beta and/or phospho-tau cause dementia, or specifically AD. Studies on post-mortem brain tissue sections of healthy controls revealed vast deposits of amyloid-beta in the brain despite a lack of symptoms [98]. Despite findings from animal models and cell culture studies, it is currently understood that the onset of dementia pathology occurs decades before the first symptoms manifest themselves [27], supporting the hypotheses that protein homeostasis mechanisms and other cellular functions begin to deteriorate long before amyloid-beta deposition and NFT formation become apparent [30,97]. Furthermore, research focus has been shifting to other cell types in addition to neurons, specifically glia and the cells of the brain vasculature, as evidence suggests that impaired communication between these cell types and neurons also promotes disease progression [27].

This very tight link of amyloid-beta and phospho-tau with dementia pathology and AD has posed major challenges for this GO annotation project. The purpose of GO is to capture the ‘normal’ physiological roles of gene products, not their roles in disease pathology. Although the impact of amyloid-beta and phospho-tau can be captured using phenotype ontologies, a combined disease and GO database that applies GO terms in the context of a pathological environment is currently missing from the resources that are available to the biomedical research communities. The question of what is ‘normal’, especially in the context of amyloid-beta and a lack of clear boundaries between ageing-related biological aspects and disease processes, still exists [98]. Therefore, the first goal of this project was to establish whether any roles of amyloid-beta, and, more importantly, its multimeric forms, could be captured using GO.

A literature search revealed that in normal physiological conditions, amyloid-beta levels are regulated by neuronal activity [99,100,101], and that amyloid-beta in turn modulates synaptic plasticity in neural circuits [66,102]. Amyloid-beta has also been shown to positively affect neuronal growth [62,76]. In addition, scientific findings have demonstrated that the effects of amyloid-beta are age-related [71,72,73,74,75]. ‘Ageing’ (GO:0007568) is a normal physiological process involving the ‘loss of functions such as resistance to disease, homeostasis, and fertility, as well as wear and tear’, i.e., aspects that are directly translatable to impairments in cellular processes underlying dementia. Based on this published evidence, we curated 18 new Complex Portal (CP) entries for physiologically occurring human, mouse, and rat amyloid-beta dimers and oligomers (Table S2), and we annotated their roles using GO. Our annotations will enable users to extract a list of the proteins known to interact with amyloid-beta. Furthermore, the detection of dysregulated amyloid-beta-interacting proteins within ‘-omics’ datasets will be facilitated.

In addition to amyloid-beta monomers, dimers, and oligomers as well as tau, 74 gene products were prioritised for GO annotation, following dementia experts’ advice, and these included proteins and protein complexes interacting with either amyloid-beta or tau (Table S1). As we take a process-focussed approach to curation, we annotate research articles fully and capture the roles of all of the gene products that were investigated and described in any given research paper. Consequently, the total number of gene products annotated in the context of amyloid-beta and tau biology was 561, of which 308 were human, i.e., far more gene products were annotated than those in the prioritised list. The majority of the annotated human gene products were proteins, but some protein complexes (including six types of amyloid-beta complexes and two of their interacting partners prioritised for annotation) and two microRNAs were also annotated as a part of this project (Table S3A). These 561 gene products have been associated with 3886 GO terms, based on published evidence; of these, 308 human gene products have been associated with 2770 GO terms (Table 1). Hence, by contributing these new ARUK-UCL annotations, we have greatly expanded the representation of dementia-relevant knowledge in GO, incorporating new GO annotations for four (gene products of *CLU*, *PICALM*, *APOE*, and *BIN1*) of the 21 gene products previously annotated as a part of the AD-focussed initiative at the University of Toronto [39]. Furthermore, prior to this project, there were no GO terms associated with either the complement receptor type 3 complex (CP-1826), or the amylin receptor 3 complex (CP-3187), which are both products of genes prioritised for annotation (Table S1). As a result of this project, there are currently 14 and 21 annotations, respectively, for these two complexes in the GO database (data from QuickGO, accessed: 30 October 2018).

Collectively, the 3886 annotations describing the normal physiological roles of the prioritised gene products interacting with either amyloid-beta or tau, as well as other proteins, complexes, and microRNAs implicated in processes impaired in AD, have greatly expanded the representation of dementia-relevant neurological processes, functions, and cellular compartments in GO. Over the past two years, increasing numbers of annotations have been included in the GO Consortium annotation files, and we have demonstrated that this improves the interpretation of AD proteomic data (Tables S7–S10). Our focus on dementia-relevant processes has contributed to this, for example, by creating ‘regulation of neuron death’ annotations to some of the prioritised proteins. By capturing published knowledge about these processes, we are providing the AD and dementia research communities with a resource that is potentially useful for diagnostic purposes and/or disease prevention. The annotation of dementia-relevant gene products involved in these early cellular processes makes knowledge available for analyses of clinical or experimental datasets related to cognition, which could help delineate the biological pathways that can be targeted for diagnostic or preventive purposes decades prior to the onset of this debilitating disorder.

### 4.2. Representing Neurobiology Using Gene Ontology

The dementia-focussed ARUK-UCL project described here and the University of Toronto GO annotation project [39] are not the only GO initiatives intended to improve the representation of the neurobiological knowledge domain in GO. Our team was previously funded to capture the knowledge relevant to Parkinson’s disease (annotations assigned by ParkinsonsUK-UCL) [40,103,104], and we have also participated in a large multi-center collaborative effort to curate published information about the synapse (annotations assigned by SynGO and SynGO-UCL) [105]. Furthermore, our interactions with other members of the GO Consortium, including biocurators of model organism resources, have been very important for the success of this project, especially in the context of GO development and revisions.

Overall, the project has resulted in the addition of 71 new GO terms to the ontology (Table S6; data from AmiGO2, accessed: 30 May 2018). These GO terms were created for the annotation of research findings involving the prioritised gene products, but many of them have also been used for manual annotation by other curation teams, as well as included in automatic annotation approaches [64]. For instance, the ARUK-UCL-contributed ‘neuron projection arborisation’ (GO:0140058) GO term has been used in a total of 90 annotations, of which 11 are manual annotations, although only three of these annotations resulted from this project (data from QuickGO, accessed: 11 October 2018). This shows that other members of the GO Consortium are also using GO terms resulting from this project for the annotation of their biological domains. This is just one example of how work resulting from one focussed GO annotation initiative aids the work of other curation teams, and thus benefits wider communities of researchers who use GO in their data analyses.

In addition to contributing new GO terms, work on this project also led to the revision of thirteen previously existing GO terms, as well as the logical relations between them (Table S6). A number of these revisions were prompted because new terms were required to annotate gene products prioritised in this project. However, the revisions were improved by additional new term requests and suggestions from biocurators at the Rat Genome Database (RGD). GO branches that were revised and enhanced through these concerted efforts organised the knowledge about descendants of the biological process ‘neuron projection development’ (GO:0031175) GO terms (Figure 4) and the cellular component ‘neuron projection’ (GO:0043005) GO terms (Figure 5). These improvements to the ontology are crucial for the accurate representation of the dementia-relevant neurobiological domain using GO. In fact, this project has resulted in 57 annotations to ‘neuron projection development’, or one of its descendant terms, and 249 annotations to ‘neuron projection’, or one of its descendants (data from QuickGO, accessed: 11 October 2018).

A close collaboration is of even greater importance when ontology revisions need to be coordinated with revisions of existing GO annotations. This was demonstrated when it became apparent that updates to the annotations associated with the GO term ‘synaptic vesicle endocytosis’ were required. ‘Synaptic vesicle endocytosis’ was originally defined as a clathrin-mediated process; however, FlyBase [106] biocurators identified that not all of the endocytic processes occurring in the synapse depend on clathrin. Prior to this, there had been a total of 97 experimentally evidenced annotations to ‘synaptic vesicle endocytosis’ assigned by 10 different curation groups. Thus, in order to reach a GO Consortium-wide consensus about whether to broaden the definition of this term to include endocytic mechanisms not requiring the involvement of clathrin, or whether to update the GO term name to ‘clathrin-dependent synaptic vesicle endocytosis’, it was crucial to first review the existing annotations to determine whether the experimental support did indeed provide evidence for clathrin mediating the endocytosis. As a result of this combined effort, and following consultations with synapse biology experts, a decision was made to broaden the definition of the existing ‘synaptic vesicle endocytosis’ GO term, and to add two more descriptive child GO terms to capture information about the specific types of synaptic endocytosis. Biocurators then updated the annotations using these new terms if there was sufficient experimental data to support this.

Overall, the new GO terms resulting from this project have broadened the representation of the neuroscience domain in GO, making a greater number of terms available to biocurators for the GO annotation of neurological concepts. In addition, ontology revisions helped to ensure the highest possible quality of this resource by reviewing existing annotations and ontology entries describing neurobiological concepts, and by ensuring that they are accurately defined and appropriately related to each other in the GO term hierarchy.

## 5. Conclusions

In order to understand the cellular processes underlying Alzheimer’s disease and other dementias, numerous transcriptomic, proteomic, and genome-wide association (GWA) studies have been conducted [2,4,5,6]. The analyses and interpretation of results from such high-throughput analyses greatly rely on functional annotation data provided by resources such as GO [10], KEGG [12], or Reactome [11], as well as on appropriate experimental design, the analysis tools used, and the settings applied. Among these, analyses performed using the latter two resources can yield informative results only if the dataset being analysed contains gene products with known functions in biological pathways. In contrast, the GO resource captures the cellular roles and locations of a higher number of gene products, even when the molecular functions of gene products are unknown. Therefore, GO is suitable for analyses of datasets that are likely to contain gene products whose specific function and biological role have not yet been fully investigated. Yet, prior to this project, the applicability of GO for the analysis of dementia-relevant neurological datasets was limited, because there had been no previous efforts to comprehensively annotate gene products with roles in this biological domain, with the exception of a project undertaken by the University of Toronto [39] that focussed only on a small group of AD risk genes.

So far, our commitment to improving the GO resource for dementia-relevant research has focussed on amyloid-beta, the microtubule-associated protein tau and its interacting partners, as described here. Our ongoing and future efforts aim to capture the biology of neuroinflammatory processes, e.g., the activation of glial cells in response to an inflammatory stimulus [107], the functional information about proteins involved in these processes, as well as knowledge about the microRNAs that regulate the expression of these proteins. As an initial focus, we have been annotating the roles of proteins involved in the biology of glial cells, primarily microglia; the lists of proteins that we have prioritised for annotation are available on our website [108]. Additionally, we have begun the GO annotation of microRNAs involved in the regulation of the expression of these proteins, primarily focussing on capturing experimentally evidenced microRNA–target interaction.

Through our systematic and focussed development of GO terms relevant to dementia and revision of previously existing neurological GO terms, as well as by detailed manual GO annotation of gene products implicated in dementia, we have enhanced the suitability of the GO resource for analysis of neurological ‘-omics’ datasets. These ongoing and future contributions to the GO resource will help provide insights into the molecular bases of dementia, thus supporting the development of treatments and of tools for early diagnosis.

All of the GO terms and annotations are freely available and can be downloaded from QuickGO [51,52,53] and AmiGO [54,55]. Information on protein complexes can be found at the Complex Portal [63,109]. We encourage scientists to become involved in the GO annotation of their own papers and/or gene products of interest. In order to contribute to GO, please contact UCL Functional Gene Annotation [108], GOA (https://www.ebi.ac.uk/GOA/contactus) [110] or the GO Consortium (http://geneontology.org/page/contributing-go) [111].

## Figures and Tables

**Table 1:** Summary of key curation statistics. (Data from QuickGO [51,52,53] and the Complex Portal [14,63]—accessed: 18 October 2018—summarised in Supplementary Tables S1–S6).

ARUK-UCL GO Annotation Project: Priorities and Curation Summary	Total	
Prioritised human gene products	84	
Prioritised human amyloid-beta-relevant gene products ^#^	50	
Prioritised human tau-relevant gene products ^##^	34	
	All species	Human
PubMed identifiers (PMIDs) curated *	226	191
GO annotations **	3886	2770
Gene products annotated **	561	308
GO annotations associated with 84 human gene products prioritised for annotation *^#^	n/a	2055
New Complex Portal (CP) entries ***	18	6

^#^ For priority amyloid-beta-relevant gene products annotations, see Table S1A; ^##^ For priority tau-relevant gene products annotations, see Table S1B; * For PMIDs annotated by ARUK-UCL, see Table S3C; ** For all of the ARUK-UCL annotations and details of gene products, see Table S3A; *^#^ For ARUK-UCL annotations to prioritised gene products, see Table S4 and filter ‘Assigned By’ A → Z to group ‘ARUK-UCL’ annotations together; *** For details about new CP details, see Table S2.

## Supplementary Materials

The following are available online at http://www.mdpi.com/2073-4425/9/12/593/s1, Table S1: Interacting partners of amyloid-beta(A) and microtubule-associated protein tau (B) prioritised for GO annotation, Table S2: Amyloid-beta monomers and/or oligomers, Table S3: All ARUK-UCL GO annotations, Table S4: All manually-curated GO annotations for the prioritised gene products (Table S1), contributed by ARUK-UCL, as well as by other groups (Data from QuickGO - accessed: 31 October 2018; filtered by database identifiers from Table S1 and evidence used in manual assertion (ECO:0000352), Table S5: Manually curated neurological GO term annotations for the prioritised gene products, contributed by ARUK-UCL, as well as by other groups (Data from QuickGO, accessed: 31 October 2018), Table S6: GO development and revisions, Table S7: Summary of four separate g:GOSt analyses of AD hippocampal datasets. Table S8: AD hippocampal protein list, Table S9: Alzheimer’s disease (AD)-relevant filter terms included in Table S7, Table S10: Number of enriched GO terms within each grouping term included in Table S7, Table S11: Summary of the number of GO terms significantly enriched in the different analyses in Table S7.

## References

[1] Van Cauwenberghe C., Van Broeckhoven C., Sleegers K. (2016). The genetic landscape of Alzheimer disease: Clinical implications and perspectives. Genet. Med..

[2] Sassi C., Nalls M.A., Ridge P.G., Gibbs J.R., Ding J., Lupton M.K., Troakes C., Lunnon K., Al-Sarraj S., Brown K.S. (2016). ABCA7 p.G215S as potential protective factor for Alzheimer’s disease. Neurobiol. Aging.

[3] Barnes D.E., Yaffe K. (2011). The projected effect of risk factor reduction on Alzheimer’s disease prevalence. Lancet Neurol..

[4] Cooper-Knock J., Kirby J., Ferraiuolo L., Heath P.R., Rattray M., Shaw P.J. (2012). Gene expression profiling in human neurodegenerative disease. Nat. Rev. Neurol..

[5] Guerreiro R., Wojtas A., Bras J., Carrasquillo M., Rogaeva E., Majounie E., Cruchaga C., Sassi C., Kauwe J.S., Younkin S. (2013). *TREM2* variants in Alzheimer’s disease. N. Engl. J. Med..

[6] Kang M.G., Byun K., Kim J.H., Park N.H., Heinsen H., Ravid R., Steinbusch H.W., Lee B., Park Y.M. (2015). Proteogenomics of the human hippocampus: The road ahead. Biochim. Biophys. Acta.

[7] Guio-Vega G.P., Forero D.A. (2017). Functional genomics of candidate genes derived from genome-wide association studies for five common neurological diseases. Int. J. Neurosci..

[8] Ebbert M.T., Ridge P.G., Kauwe J.S. (2015). Bridging the gap between statistical and biological epistasis in Alzheimer’s disease. Biomed. Res. Int..

[9] Cambiaghi A., Ferrario M., Masseroli M. (2017). Analysis of metabolomic data: Tools, current strategies and future challenges for omics data integration. Brief Bioinform..

[10] Ashburner M., Ball C.A., Blake J.A., Botstein D., Butler H., Cherry J.M., Davis A.P., Dolinski K., Dwight S.S., Eppig J.T. (2000). Gene ontology: Tool for the unification of biology. The Gene Ontology Consortium. Nat. Genet..

[11] Fabregat A., Sidiropoulos K., Garapati P., Gillespie M., Hausmann K., Haw R., Jassal B., Jupe S., Korninger F., McKay S. (2016). The Reactome pathway knowledgebase. Nucleic Acids Res..

[12] Kanehisa M., Sato Y., Kawashima M., Furumichi M., Tanabe M. (2016). KEGG as a reference resource for gene and protein annotation. Nucleic Acids Res..

[13] Orchard S., Ammari M., Aranda B., Breuza L., Briganti L., Broackes-Carter F., Campbell N.H., Chavali G., Chen C., del-Toro N. (2014). The MIntAct project—IntAct as a common curation platform for 11 molecular interaction databases. Nucleic Acids Res..

[14] Meldal B.H.M., Bye A.J.H., Gajdos L., Hammerova Z., Horackova A., Melicher F., Perfetto L., Pokorny D., Lopez M.R., Turkova A. (2018). Complex Portal 2018: Extended content and enhanced visualization tools for macromolecular complexes. Nucleic Acids Res..

[15] Reimand J., Arak T., Adler P., Kolberg L., Reisberg S., Peterson H., Vilo J. (2016). g:Profiler—A web server for functional interpretation of gene lists (2016 update). Nucleic Acids Res..

[16] Richardson J.E., Bult C.J. (2015). Visual annotation display (VLAD): A tool for finding functional themes in lists of genes. Mamm. Genome.

[17] Huang D.W., Sherman B.T., Lempicki R.A. (2009). Systematic and integrative analysis of large gene lists using DAVID Bioinformatics Resources. Nat. Protoc..

[18] Alam-Faruque Y., Huntley R.P., Khodiyar V.K., Camon E.B., Dimmer E.C., Sawford T., Martin M.J., O’Donovan C., Talmud P.J., Scambler P. (2011). The impact of focused Gene Ontology curation of specific mammalian systems. PLoS ONE.

[19] Patel S., Roncaglia P., Lovering R.C. (2015). Using Gene Ontology to describe the role of the neurexin-neuroligin-SHANK complex in human, mouse and rat and its relevance to autism. BMC Bioinform..

[20] Gray K.A., Yates B., Seal R.L., Wright M.W., Bruford E.A. (2015). Genenames.org: The HGNC resources in 2015. Nucleic Acids Res..

[21] Masino A.J., Dechene E.T., Dulik M.C., Wilkens A., Spinner N.B., Krantz I.D., Pennington J.W., Robinson P.N., White P.S. (2014). Clinical phenotype-based gene prioritization: An initial study using semantic similarity and the human phenotype ontology. BMC Bioinform..

[22] Kametani F., Hasegawa M. (2018). Reconsideration of amyloid hypothesis and tau hypothesis in Alzheimer’s Disease. Front. Neurosci..

[23] Hardy J., Allsop D. (1991). Amyloid deposition as the central event in the aetiology of Alzheimer’s disease. Trends Pharmacol. Sci..

[24] Goedert M. (1996). Tau protein and the neurofibrillary pathology of Alzheimer’s disease. Ann. N. Y. Acad. Sci..

[25] Goedert M. (1993). Tau protein and the neurofibrillary pathology of Alzheimer’s disease. Trends Neurosci..

[26] Selkoe D.J., Hardy J. (2016). The amyloid hypothesis of Alzheimer’s disease at 25 years. EMBO Mol. Med..

[27] De Strooper B., Karran E. (2016). The cellular phase of Alzheimer’s disease. Cell.

[28] Schott J.M., Revesz T. (2013). Inflammation in Alzheimer’s disease: Insights from immunotherapy. Brain.

[29] Nelson A.R., Sweeney M.D., Sagare A.P., Zlokovic B.V. (2016). Neurovascular dysfunction and neurodegeneration in dementia and Alzheimer’s disease. Biochim. Biophys. Acta.

[30] Kundra R., Ciryam P., Morimoto R.I., Dobson C.M., Vendruscolo M. (2017). Protein homeostasis of a metastable subproteome associated with Alzheimer’s disease. Proc. Natl. Acad. Sci. USA.

[31] Cuyvers E., Sleegers K. (2016). Genetic variations underlying Alzheimer’s disease: Evidence from genome-wide association studies and beyond. Lancet Neurol..

[32] Shen L., Jia J. (2016). An Overview of genome-wide association studies in Alzheimer’s disease. Neurosci. Bull..

[33] De Matos M.R., Ferreira C., Herukka S.K., Soininen H., Janeiro A., Santana I., Baldeiras I., Almeida M.R., Lleo A., Dols-Icardo O. (2018). Quantitative genetics validates previous genetic variants and identifies novel genetic players influencing Alzheimer’s disease cerebrospinal fluid biomarkers. J. Alzheimers Dis..

[34] Abu-Rumeileh S., Mometto N., Bartoletti-Stella A., Polischi B., Oppi F., Poda R., Stanzani-Maserati M., Cortelli P., Liguori R., Capellari S., Parchi P. (2018). Cerebrospinal fluid biomarkers in patients with frontotemporal dementia spectrum: A single-center study. J. Alzheimers Dis..

[35] Verheijen J., Sleegers K. (2018). Understanding Alzheimer disease at the interface between genetics and transcriptomics. Trends Genet..

[36] Espuny-Camacho I., Arranz A.M., Fiers M., Snellinx A., Ando K., Munck S., Bonnefont J., Lambot L., Corthout N., Omodho L. (2017). Hallmarks of Alzheimer’s disease in stem-cell-derived human neurons transplanted into mouse brain. Neuron.

[37] Xu J., Patassini S., Rustogi N., Riba-Garcia I., Hale B.D., Phillips A.M., Waldvogel H., Haines R., Bradbury P., Stevens A. (2018). Regional protein expression in human Alzheimer’s brain correlates with disease severity. bioRxiv.

[38] Kunkle B.W., Grenier-Boley B., Sims R., Bis J.C., Naj A.C., Boland A., Vronskaya M., van der Lee S.J., Amlie-Wolf A., Bellenguez C. (2018). Meta-analysis of genetic association with diagnosed Alzheimer’s disease identifies novel risk loci and implicates Abeta, Tau, immunity and lipid processing. bioRxiv.

[39] Patel S., Park M. (2016). Gene prioritization for imaging genetics studies using gene ontology and a stratified false discovery rate approach. Front. Neuroinform..

[40] Denny P., Feuermann M., Hill D.P., Lovering R.C., Plun-Favreau H., Roncaglia P. (2018). Exploring autophagy with Gene Ontology. Autophagy.

[41] Jarosz-Griffiths H.H., Noble E., Rushworth J.V., Hooper N.M. (2016). Amyloid-beta receptors: The good, the bad, and the prion protein. J. Biol. Chem..

[42] Guo T., Noble W., Hanger D.P. (2017). Roles of tau protein in health and disease. Acta Neuropathol..

[43] The Gene Ontology Consortium (2017). Expansion of the Gene Ontology knowledgebase and resources. Nucleic Acids Res..

[44] NCBI PubMed. https://www.ncbi.nlm.nih.gov/pubmed/.

[45] Balakrishnan R., Harris M.A., Huntley R., Van Auken K., Cherry J.M. (2013). A guide to best practices for Gene Ontology (GO) manual annotation. Database.

[46] Huntley R.P., Harris M.A., Alam-Faruque Y., Blake J.A., Carbon S., Dietze H., Dimmer E.C., Foulger R.E., Hill D.P., Khodiyar V.K. (2014). A method for increasing expressivity of Gene Ontology annotations using a compositional approach. BMC Bioinform..

[47] Pundir S., Martin M.J., O’Donovan C., UniProt Consortium (2016). UniProt tools. Curr. Protoc. Bioinform..

[48] The RNAcentral Constortium (2018). RNAcentral: A hub of information for non-coding RNA sequences. Nucleic Acids Res..

[49] Gene Ontology Consortium (2016). Gene Ontology Evidence Code Documentation. http://www.geneontology.org/page/guide-go-evidence-codes.

[50] Huntley R.P., Sawford T., Mutowo-Meullenet P., Shypitsyna A., Bonilla C., Martin M.J., O’Donovan C. (2015). The GOA database: Gene Ontology annotation updates for 2015. Nucleic Acids Res..

[51] Huntley R.P., Binns D., Dimmer E., Barrell D., O’Donovan C., Apweiler R. (2009). QuickGO: A user tutorial for the web-based Gene Ontology browser. Database.

[52] Binns D., Dimmer E., Huntley R., Barrell D., O’Donovan C., Apweiler R. (2009). QuickGO: A web-based tool for Gene Ontology searching. Bioinformatics.

[53] EMBL-EBI, QuickGO. https://www.ebi.ac.uk/QuickGO/.

[54] Carbon S., Ireland A., Mungall C.J., Shu S., Marshall B., Lewis S. (2009). AmiGO: Online access to ontology and annotation data. Bioinformatics.

[55] AmiGO 2. http://amigo.geneontology.org/amigo/landing.

[56] Brown G.R., Hem V., Katz K.S., Ovetsky M., Wallin C., Ermolaeva O., Tolstoy I., Tatusova T., Pruitt K.D., Maglott D.R., Murphy T.D. (2015). Gene: A gene-centered information resource at NCBI. Nucleic Acids Res..

[57] Newman V., Moore B., Sparrow H., Perry E. (2018). The Ensembl Genome Browser: Strategies for accessing eukaryotic genome data. Methods Mol. Biol..

[58] Griffiths-Jones S., Grocock R.J., van Dongen S., Bateman A., Enright A.J. (2006). miRBase: microRNA sequences, targets and gene nomenclature. Nucleic Acids Res..

[59] Kibbe W.A., Arze C., Felix V., Mitraka E., Bolton E., Fu G., Mungall C.J., Binder J.X., Malone J. (2015). Disease Ontology 2015 update: An expanded and updated database of human diseases for linking biomedical knowledge through disease data. Nucleic Acids Res..

[60] GitHub. https://github.com/.

[61] AmiGO 2. http://amigo.geneontology.org/amigo/landing.

[62] Lopez-Toledano M.A., Shelanski M.L. (2004). Neurogenic effect of beta-amyloid peptide in the development of neural stem cells. J. Neurosci..

[63] EMBL-EBI Complex Portal. https://www.ebi.ac.uk/complexportal/home.

[64] Huntley R.P., Sawford T., Martin M.J., O’Donovan C. (2014). Understanding how and why the Gene Ontology and its annotations evolve: The GO within UniProt. Gigascience.

[65] Citri A., Malenka R.C. (2008). Synaptic plasticity: Multiple forms, functions, and mechanisms. Neuropsychopharmacology.

[66] Puzzo D., Privitera L., Leznik E., Fa M., Staniszewski A., Palmeri A., Arancio O. (2008). Picomolar amyloid-beta positively modulates synaptic plasticity and memory in hippocampus. J. Neurosci..

[67] Perez-Nievas B.G., Stein T.D., Tai H.C., Dols-Icardo O., Scotton T.C., Barroeta-Espar I., Fernandez-Carballo L., de Munain E.L., Perez J., Marquie M. (2013). Dissecting phenotypic traits linked to human resilience to Alzheimer’s pathology. Brain.

[68] Shimoyama M., De Pons J., Hayman G.T., Laulederkind S.J., Liu W., Nigam R., Petri V., Smith J.R., Tutaj M., Wang S.J. (2015). The Rat Genome Database 2015: Genomic, phenotypic and environmental variations and disease. Nucleic Acids Res..

[69] Milosevic I. (2018). Revisiting the role of clathrin-mediated endoytosis in synaptic vesicle recycling. Front. Cell. Neurosci..

[70] Gan Q., Watanabe S. (2018). Synaptic vesicle endocytosis in different model systems. Front. Cell. Neurosci..

[71] Chen G., Chen K.S., Knox J., Inglis J., Bernard A., Martin S.J., Justice A., McConlogue L., Games D., Freedman S.B. (2000). A learning deficit related to age and beta-amyloid plaques in a mouse model of Alzheimer’s disease. Nature.

[72] Janus C., Pearson J., McLaurin J., Mathews P.M., Jiang Y., Schmidt S.D., Chishti M.A., Horne P., Heslin D., French J. (2000). A beta peptide immunization reduces behavioural impairment and plaques in a model of Alzheimer’s disease. Nature.

[73] Westerman M.A., Cooper-Blacketer D., Mariash A., Kotilinek L., Kawarabayashi T., Younkin L.H., Carlson G.A., Younkin S.G., Ashe K.H. (2002). The relationship between Abeta and memory in the Tg2576 mouse model of Alzheimer’s disease. J. Neurosci..

[74] Takeda S., Hashimoto T., Roe A.D., Hori Y., Spires-Jones T.L., Hyman B.T. (2013). Brain interstitial oligomeric amyloid-beta increases with age and is resistant to clearance from brain in a mouse model of Alzheimer’s disease. FASEB J..

[75] Shankar G.M., Leissring M.A., Adame A., Sun X., Spooner E., Masliah E., Selkoe D.J., Lemere C.A., Walsh D.M. (2009). Biochemical and immunohistochemical analysis of an Alzheimer’s disease mouse model reveals the presence of multiple cerebral Abeta assembly forms throughout life. Neurobiol. Dis..

[76] Yankner B.A., Duffy L.K., Kirschner D.A. (1990). Neurotrophic and neurotoxic effects of amyloid-beta protein: Reversal by tachykinin neuropeptides. Science.

[77] Barghorn S., Nimmrich V., Striebinger A., Krantz C., Keller P., Janson B., Bahr M., Schmidt M., Bitner R.S., Harlan J. (2005). Globular amyloid-beta-peptide oligomer—A homogenous and stable neuropathological protein in Alzheimer’s disease. J. Neurochem..

[78] Nimmrich V., Grimm C., Draguhn A., Barghorn S., Lehmann A., Schoemaker H., Hillen H., Gross G., Ebert U., Bruehl C. (2008). Amyloid-β oligomers (A-β_1-42_ globulomer) suppress spontaneous synaptic activity by inhibition of P/Q-type calcium currents. J. Neurosci..

[79] Noguchi A., Matsumura S., Dezawa M., Tada M., Yanazawa M., Ito A., Akioka M., Kikuchi S., Sato M., Ideno S. (2009). Isolation and characterization of patient-derived, toxic, high mass amyloid-β-protein (A-β) assembly from Alzheimer disease brains. J. Biol. Chem..

[80] Ohnishi T., Yanazawa M., Sasahara T., Kitamura Y., Hiroaki H., Fukazawa Y., Kii I., Nishiyama T., Kakita A., Takeda H. (2015). Na, K-ATPase α3 is a death target of Alzheimer patient amyloid-β assembly. Proc. Natl. Acad. Sci. USA.

[81] Malhotra A., Younesi E., Gundel M., Muller B., Heneka M.T., Hofmann-Apitius M. (2014). ADO: A disease ontology representing the domain knowledge specific to Alzheimer’s disease. Alzheimers Dement..

[82] Drame K., Diallo G., Delva F., Dartigues J.F., Mouillet E., Salamon R., Mougin F. (2014). Reuse of termino-ontological resources and text corpora for building a multilingual domain ontology: An application to Alzheimer’s disease. J. Biomed. Inform..

[83] Refolo L.M., Snyder H., Liggins C., Ryan L., Silverberg N., Petanceska S., Carrillo M.C. (2012). Common Alzheimer’s disease research ontology: National institute on aging and Alzheimer’s association collaborative project. Alzheimers Dement..

[84] Liggins C., Snyder H.M., Silverberg N., Petanceska S., Refolo L.M., Ryan L., Carrillo M.C. (2014). International Alzheimer’s disease research portfolio (IADRP) aims to capture global Alzheimer’s disease research funding. Alzheimers Dement..

[85] Zhang X., Hu B., Ma X., Moore P., Chen J. (2014). Ontology driven decision support for the diagnosis of mild cognitive impairment. Comput. Methods Prog. Biomed..

[86] Lovering R.C., Roncaglia P., Howe D.G., Laulederkind S.J.F., Khodiyar V.K., Berardini T.Z., Tweedie S., Foulger R.E., Osumi-Sutherland D., Campbell N.H. (2018). Improving Interpretation of cardiac phenotypes and enhancing discovery with expanded knowledge in the gene ontology. Circ. Genom Precis Med..

[87] Ferrari R., Grassi M., Salvi E., Borroni B., Palluzzi F., Pepe D., D’avila F., Padovani A., Archetti S., Rainero I. (2015). A genome-wide screening and SNPs-to-genes approach to identify novel genetic risk factors associated with frontotemporal dementia. Neurobiol. Aging.

[88] Welton J.L., Loveless S., Stone T., Von Ruhland C., Robertson N.P., Clayton A. (2017). Cerebrospinal fluid extracellular vesicle enrichment for protein biomarker discovery in neurological disease; multiple sclerosis. J. Extracell. Vesicles.

[89] Hirsch T., Rothoeft T., Teig N., Bauer J.W., Pellegrini G., De Rosa L., Scaglione D., Reichelt J., Klausegger A., Kneisz D. (2017). Regeneration of the entire human epidermis using transgenic stem cells. Nature.

[90] Ittner L.M., Ke Y.D., Delerue F., Bi M., Gladbach A., Van Eersel J., Wolfing H., Chieng B.C., Christie M.J., Napier I.A. (2010). Dendritic function of tau mediates amyloid-beta toxicity in Alzheimer’s disease mouse models. Cell.

[91] Iqbal K., Liu F., Gong C.X., Grundke-Iqbal I. (2010). Tau in Alzheimer disease and related tauopathies. Curr. Alzheimer Res..

[92] Butterfield D.A., Boyd-Kimball D. (2004). Amyloid-beta-peptide(1-42) contributes to the oxidative stress and neurodegeneration found in Alzheimer disease brain. Brain Pathol..

[93] Mudher A., Colin M., Dujardin S., Medina M., Dewachter I., Alavi Naini S.M., Mandelkow E.M., Mandelkow E., Buee L., Goedert M. (2017). What is the evidence that tau pathology spreads through prion-like propagation?. Acta Neuropathol. Commun..

[94] Kaufman S.K., Thomas T.L., Del Tredici K., Braak H., Diamond M.I. (2017). Characterization of tau prion seeding activity and strains from formaldehyde-fixed tissue. Acta Neuropathol. Commun..

[95] Kaufman S.K., Del Tredici K., Thomas T.L., Braak H., Diamond M.I. (2018). Tau seeding activity begins in the transentorhinal/entorhinal regions and anticipates phospho-tau pathology in Alzheimer’s disease and PART. Acta Neuropathol..

[96] DeVos S.L., Corjuc B.T., Oakley D.H., Nobuhara C.K., Bannon R.N., Chase A., Commins C., Gonzalez J.A., Dooley P.M., Frosch M.P. (2018). Synaptic tau seeding precedes tau pathology in human Alzheimer’s disease brain. Front. Neurosci..

[97] Hyttinen J.M., Amadio M., Viiri J., Pascale A., Salminen A., Kaarniranta K. (2014). Clearance of misfolded and aggregated proteins by aggrephagy and implications for aggregation diseases. Ageing Res. Rev..

[98] Rodrigue K.M., Kennedy K.M., Park D.C. (2009). Beta-amyloid deposition and the aging brain. Neuropsychol. Rev..

[99] Kamenetz F., Tomita T., Hsieh H., Seabrook G., Borchelt D., Iwatsubo T., Sisodia S., Malinow R. (2003). APP processing and synaptic function. Neuron.

[100] Cirrito J.R., Yamada K.A., Finn M.B., Sloviter R.S., Bales K.R., May P.C., Schoepp D.D., Paul S.M., Mennerick S., Holtzman D.M. (2005). Synaptic activity regulates interstitial fluid amyloid-beta levels in vivo. Neuron.

[101] Li X., Uemura K., Hashimoto T., Nasser-Ghodsi N., Arimon M., Lill C.M., Palazzolo I., Krainc D., Hyman B.T., Berezovska O. (2013). Neuronal activity and secreted amyloid-beta lead to altered amyloid-beta precursor protein and presenilin 1 interactions. Neurobiol. Dis..

[102] Cao L., Schrank B.R., Rodriguez S., Benz E.G., Moulia T.W., Rickenbacher G.T., Gomez A.C., Levites Y., Edwards S.R., Golde T.E. (2012). Abeta alters the connectivity of olfactory neurons in the absence of amyloid plaques in vivo. Nat. Commun..

[103] Denny P., Feuermann M., Hill D.P., Roncaglia P., Lovering R.C. (2016). Exploring autophagy with Gene Ontology. F1000Research (Poster).

[104] Foulger R.E., Denny P., Hardy J., Martin M.J., Sawford T., Lovering R.C. (2016). Using the Gene Ontology to annotate key players in Parkinson’s disease. Neuroinformatics.

[105] (2018). Gene Ontology Consortium, SynGO—Synapse Biology. http://www.geneontology.org/page/syngo-synapse-biology.

[106] Thurmond J., Goodman J.L., Strelets V.B., Attrill H., Gramates L.S., Marygold S.J., Matthews B.B., Millburn G., Antonazzo G., Trovisco V. (2018). FlyBase 2.0: the next generation. Nucleic Acids Res..

[107] Li Q., Barres B.A. (2018). Microglia and macrophages in brain homeostasis and disease. Nat. Rev. Immunol..

[108] UCL Functional Gene Annotation, Neurological Gene Ontology. https://www.ucl.ac.uk/functional-gene-annotation/neurological.

[109] Meldal B.H., Forner-Martinez O., Costanzo M.C., Dana J., Demeter J., Dumousseau M., Dwight S.S., Gaulton A., Licata L., Melidoni A.N. (2015). The complex portal—an encyclopaedia of macromolecular complexes. Nucleic Acids Res..

[110] GOA Contact Us. https://www.ebi.ac.uk/GOA/contactus.

[111] Contributing to GO. http://geneontology.org/page/contributing-go.

